# The importance of fetal gender in intrauterine growth restriction

**Published:** 2013-03-25

**Authors:** L Radulescu, D Ferechide, F Popa

**Affiliations:** * “Alfred Rusescu" Institute for Mother and Child, Bucharest; ** “Carol Davila" University of Medicine and Pharmacy, Bucharest

**Keywords:** fetal gender, intrauterine growth restriction, IUGR

## Abstract

One of the most important causes of perinatal mortality and morbidity complicating significant percentage pregnancies is intrauterine growth restriction (IUGR).

Fetal growth restriction is the main cause of intrauterine fetal death and the second leading cause of death in the neonatal period.

Numerous studies in different populations reveal an association between intrauterine growth restriction and perinatal and postnatal developments, which differ according to the sex of newborns with intrauterine growth restriction. However, the mechanisms of intrauterine programming, the critical time necessary to cause injury and involvement of other factors are unclear and although several authors’ opinions differ, it seems that females are more likely to develop intrauterine growth restriction.

**Abbreviations:** IUGR=intrauterine growth restriction

## Introduction

One area of concern for society and for the medical system is the fetal biological qualities because of the consequences they have on the welfare of future children and adults.

 One of the most important causes of perinatal mortality and morbidity that complicates a significant percentage of the number of pregnancies is intrauterine growth restriction (IUGR).

 Fetal growth restriction is the inability of the fetus to achieve its genetically determined potential dimension and represents the leading cause of intrauterine fetal death (60-70%) [**1,2**] and the second cause of death in the neonatal period - 41% [**3**].

**Fig. 1:**
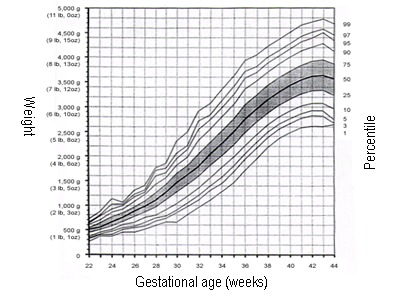
Fetal growth and weight percentiles for gestational age

 Fetal growth restriction (early - hypoplastic and late - hypotrophic) is an abnormal fetal growth and development, defined as fetal weight below the 10th percentile (or below 2 SD) than the average for gestational age. Cutoff weight is 2500g (5 lb 8oz) [**4-8**] (**Fig. 1**).

 In order to evaluate the effect of fetal sex on pregnancy outcome N. Melamed et al. conducted a retrospective study on 66.387 (34.367 to 51.8% male fetuses and 32.020 to 48.2% female fetuses) singleton pregnancies over a period of 11 years. They concluded that although the incidence of preterm delivery and cesarean section was higher for male, female fetuses were more likely to develop intrauterine growth restriction [**9**].

 According to the study conducted by Quinones et al. the inability of the fetus to achieve its genetically determined weight, regardless of gender, has a negative impact on the fetal morbidity and mortality. The results led to the idea that fetal sex is not an independent risk factor for perinatal development in the presence IUGR and that gender required the presence of other factors as well [**10**].

 However, other authors have concluded that the male sex is an independent risk factor for pregnancy poor outcome, although the IUGR ultrasound diagnostic rate was higher in female fetuses and the indication of cesarean delivery with the ending of labor was identified more frequently in patients with male fetuses [**11**].

## Conclusions

The general concept that male fetuses have a lower clinical performance than females apparently does not apply in the case of intrauterine growth restriction. Although opinions are divided, the scales are tipped by females, who are more prone to develop RCIU; but studies and further investigations are needed to determine the mechanisms underlying this association to our understanding the pathophysiology of pregnancy complications and to establish effective methods of prevention, diagnostic and therapeutic strategies for RCIU, perhaps affecting the health of the future generations.

 Acknowledgement 

 This paper is supported by the Sectoral Operational Programme Human Resources Development (SOP HRD) 2007-2013, financed from the European Social Fund and by the Romanian Government under the contract number POSDRU/107/1.5/S/82839.
